# Salivary cortisol response to psychosocial stress in patients with first-episode psychosis

**DOI:** 10.3325/cmj.2021.62.80

**Published:** 2021-02

**Authors:** Linda Rossini Gajšak, Željka Vogrinc, Mirela Čelić, Ružić, Dina Bošnjak, Kuharić, Marija Bošković, Ana Koričančić, Makar, Porin Makarić, Vesna Ermakora, Ivana Kekin, Žarko Bajić, Martina Rojnić Kuzman

**Affiliations:** 1 Dr Ivan Barbot Neuropsychiatric Hospital, Popovača, Croatia; 2 Department of Laboratory Diagnostics, University Hospital Center Zagreb, Zagreb, Croatia; 3 Sveti Ivan Psychiatric Hospital, Zagreb, Croatia; 4 Vrapče University Psychiatric Hospital, Zagreb, Croatia; 5 Department of Psychiatry, University Hospital Center Zagreb, Zagreb, Croatia; 6 University of Zagreb School of Medicine, Zagreb, Croatia

## Abstract

**Aim:**

To analyze the difference in the salivary cortisol response to psychosocial stress between the patients with the first episode of psychosis (FEP) and the control group.

**Methods:**

We performed a cross-sectional analysis of the baseline measurements of a prospective cohort study conducted from 2015 to 2018 at two Croatian psychiatric hospitals. The study consecutively enrolled 53 patients diagnosed with FEP and 63 healthy controls. The primary outcome was the difference in the changes of salivary cortisol concentration during the stress test. The secondary outcome was the difference in the baseline levels of salivary cortisol between patients with FEP and controls. The tertiary outcome were the correlations of salivary cortisol levels with the results of the Positive and Negative Syndrome Scale for Schizophrenia, Rosenberg Self-Esteem Scale, and the International Personality Item Pool.

**Results:**

Patients with FEP had significantly higher baseline salivary cortisol than controls, but their salivary cortisol increased significantly less during the stress test.

**Conclusion:**

Patients with FEP respond differently to stressful stimuli than controls, as shown by the increased baseline salivary cortisol and blunted cortisol response, possibly indicating a greater vulnerability to psychosocial stress.

Schizophrenia is one of the most complex psychiatric disorders (1), and in most cases, a long-term condition characterized by alternating periods of acute psychosis and remission. The first episode of psychosis (FEP) is usually preceded by a prodromal phase with non-specific symptoms, followed by psychotic symptoms pertaining to five dimensions: positive, negative, affective, cognitive, and psychomotor. While the pathogenesis is largely unknown, different streams of research consistently show that schizophrenia results from a complex gene-environment interaction (2), in line with the stress-diathesis model of schizophrenia etiology (3). According to this model, genetic and environmental factors lead to vulnerability to schizophrenia (2), and schizophrenia occurs when the vulnerable person experiences “enough” stress (4). For the majority of people, significant distress is associated with major stressors, eg, death in the family, serious illness, or separation (5). However, in persons prone to psychosis significant distress is caused even by minor stressors, also known as daily hassles (6).

Response to stress involves the activation of the hypothalamic-pituitary-adrenal axis (HPA): the hypothalamus secretes corticotropin-releasing factor (CRF), which stimulates the secretion of adrenocorticotropic hormone (ACTH) from the front pituitary gland. ACTH then stimulates the release of cortisol from the adrenal gland, causing the cascading effect of several bodily systems (immune, neuroendocrine, inflammatory response to the organism, etc) (7). In the central nervous system, increased cortisol levels can lead to the sensitization of dopaminergic response to stress, which can lead to an excessive dopamine release and psychotic symptoms (6). Indeed, various indicators of altered stress response have been confirmed in the population at risk for psychosis compared with healthy population (8,9).

While it has been suggested that persons with schizophrenia show a blunted cortisol response following experimentally induced psychosocial stress (10,11), these findings should be further elucidated considering the heterogeneous research results. Some of these discrepancies may arise from the heterogeneity of the samples and different confounding factors but also from different stress response across illness types and stages (12).

Generally, persons with FEP show higher basal cortisol values than the healthy population (8,13,14), a finding that possibly indicates their increased basic HPA hyperactivity, which contributes to their higher vulnerability to stressors. HPA axis hyperactivity indicated by elevated basal cortisol levels in individuals with psychosis is expected to also produce a higher acute elevation of cortisol concentrations in response to acute stressful situation (11). However, studies on FEP are not uniform in their findings (5-7). A small study with eleven medication-naive patients found an attenuated response of cortisol to psychosocial stress (15). Furthermore, Pruessner et al (16) reported significantly lower cortisol levels and systolic blood pressure during the psychosocial stress task in an ultra-high-risk group compared with controls; the lower cortisol levels were associated with higher self-rated stress in the past year. The authors suggested that in these patients attenuated stress response reflects the vulnerability to stressors. A recent study among patients with FEP obtained contrary results, ie, lower salivary cortisol levels at baseline but no difference in the cortisol response during psychosocial stress challenge test compared with healthy controls (17). Another study found no differences in baseline salivary cortisol levels between patients with FEP and controls, but a blunted cortisol response to stressors in FEP, both on and off medication, compared with controls (18). Finally, individuals with schizophrenia have shown lower cortisol levels both in anticipation and after exposure to social stress compared with controls, even though there were no differences in cortisol production rate (19).

Of note, people with posttraumatic stress syndrome (20) and those with high anxiety traits (21) showed higher salivary baseline cortisol, but a blunted response to psychosocial stress compared with healthy controls (22), which suggests a deficient acute neuroendocrine stress response.

Thus, the aim of our study was to assess the difference in the salivary cortisol response to the psychosocial stress between the patients with FEP and healthy controls. We enrolled only patients with FEP who were homogeneous in age, duration of illness, phase of illness, and with minimal exposure to medication to limit some of the major confounding factors from other studies. We hypothesized that patients with FEP showed higher baseline salivary cortisol levels, which indicates increased basic HPA hyperactivity, and a lower cortisol increase in response to psychosocial stressor compared with HC, as an indicator of an aberrant response to psychosocial stress. Our exploratory aim was to analyze the correlation of other clinical factors, including psychopathology, personality traits, and stressful life events with salivary cortisol levels.

## PATIENTS AND METHODS

### Study design

We performed this cross-sectional analysis on a subset of patients enrolled to a larger prospective cohort study described elsewhere (1). The larger prospective cohort study lasted from October 1, 2015 to February 31, 2018, and this analysis was performed on the patients enrolled from March 1, 2015 to December 31, 2017 only at the Department of Psychiatry, Zagreb University Hospital Centre, and Dr Ivan Barbot Neuropsychiatric Hospital, and not in other prospective cohort study centers. The study protocol was approved by the Ethics Committee of Zagreb University Hospital Centre and the Ethics Committee of Dr Ivan Barbot Neuropsychiatric Hospital. Participants signed the informed consent form. Their anonymity was protected by keeping the informed consents forms separately from the data collection instruments. We performed the study in accordance with World Medical Association Declaration of Helsinki of 1975, as revised in 2013 (23).

### Study population

The target population were patients of both sexes, 18 to 45 years old, diagnosed with FEP (codes F20, F23, F29) according to the International Classification of Diseases – 10th Revision (24). We enrolled only patients with FEP who were homogenous in age, duration of illness, phase of illness, and with minimal exposure to medication to limit some of the major confounding factors from other studies. The diagnosis was made by two experienced psychiatrists, MRK and MCR. The exclusion criteria for patients were childhood mental disorders that may present with psychosis (eg, autism), neurological disorders, moderate or severe mental retardation, and addiction to alcohol and psychoactive substances in comorbidity, pregnancy, lactation, and the use of drugs that may cause psychotic reactions for both patients and healthy controls.

All patients received standard care provided in the mentioned hospitals (treatment as usual), with medication indicated by the treating psychiatrists. The core of the hospital treatment was pharmacotherapy, based on available second- and first-generation antipsychotics (aripiprazole, clozapine, olanzapine, quetiapine, risperidone, ziprasidone, haloperidol, fluphenazine) applied orally or as long acting injectables (LAIs, for aripiprazole, olanzapine, paliperidone, risperidone, and first-generation antipsychotics). Sedatives, mood stabilizers, and antidepressants were allowed according to the clinician's indication (Table 1). After reaching the subacute phase of psychosis (usually in the first few weeks of medication treatment), patients were discharged from hospital, and their medication at discharge was recorded. The assessments were performed once the patients’ clinical condition allowed it (for the majority in the sixth-eighth week of treatment).

**Table 1:** Characteristics of participants with first-episode psychosis (FEP) and controls. Data are shown as count and percentage unless otherwise indicated*

	FEP group (n = 53)		Control group (n = 63)	
Female sex	21	(39.6)	34	(54.0)
Age, median (IQR)	25	(23-29)	22	(21-25)
University education	10	(18.9)	18	(28.6)
Being employed	13	(24.5)	19	(30.2)
Smoking tobacco	22	(41.5)	11	(17.5)
Antipsychotics				
monotherapy	33	(62.3)		
combination therapy	20	(37.7)		
Generation†				
first	15	(28.3)		
second	47	(88.7)		
Way of application				
oral	48	(90.6)		
LAI	7	(13.2)		
Benzodiazepines	23	(43.4)		
Mood stabilizers	11	(20.8)		
Antidepressants	10	(18.9)		
Anticholinergics	9	(17.0)		
Symptoms severity, mean (SD)				
PANSS total score	96	(22.4)		
positive symptoms	23	(8.5)		
negative symptoms	23	(7.0)		
general symptoms	50	(9.9)		

*Abbreviations: FEP – first episode psychosis; LAI – long-acting injectable; IQR – interquartile range, PANSS – The Positive and Negative Syndrome Scale for Schizophrenia.

†The sum exceeds 100% due to the combination therapies.

The control group included people of both sexes, 18 to 45 years old, with a negative history of psychotic disorders and other previously stated exclusion criteria. They were selected from a convenience sample of hospital staff and medical students. All included participants were interviewed by the study researchers upon study inclusion. The control group was recruited at the same time as the FEP cohort.

### Assessment of participants

All participants completed the Rosenberg Self-Esteem Scale (SES) (25) and the International Personality Item Pool (IPIP) (26). Psychopathology assessment with the Positive and Negative Syndrome Scale for Schizophrenia (PANSS) scale (27) was conducted by experienced raters MRK and MCR.

### The protocol for the stress paradigm

We used a modification of the Montreal Imaging Stress Task (28) and the Trier's Social Stress Test (TSST) (29,30). The combination of public speaking and cognitive task robustly increases cortisol (29), probably as a result of their association with social evaluative threat and uncontrollability, two important characteristics of psychological stressors (31). All participants refrained from eating, drinking, and smoking one hour before the test session. Saliva samples were collected at five time points during the test to assess salivary cortisol levels, as described in our published research protocol (1). Testing sessions took place from 13:00 to 16:00, to minimize the impact of diurnal rhythm on the cortisol response. Participants were informed about the nature of the task after baseline saliva samples had been collected. Saliva was collected using pre-labeled Salivettes^®^ (Sarstedt, Nümbrecht, Germany) on the day of psychosocial stress testing.

Salivary cortisol was measured at the Department of Laboratory Diagnostics Zagreb University Hospital Centre (1) with fully automated analyzer Roche Cobas c6000, using electrochemiluminescence immunoassay (32). The saliva samples were collected according to a previously described protocol (33).

### Statistical analysis

The needed sample size was estimated before the start of data collection based on a pilot study performed with 14 FEP patients and 17 participants with no psychotic symptoms. These participants were not included in the main study sample. The final sample of 54 in each group was needed to detect a small effect size of partial eta squared coefficient (η2) = 0.03 at two-tail *P* < 0.05 with 0.80 power when analyzing the interaction of time during the stress test and the study group by a mixed, between-within subjects analysis of covariance, and the minimum expected correlation between repeated measurements of Pearson r ≥0.60, which was obtained in the pilot study. We calculated the needed sample size using G*Power, version 3.1.9.2.

The primary outcome was the difference between FEP patients and controls in the changes of salivary cortisol concentration during the stress test. The presumed cortisol increase was operationalized as the difference between the measurement after the three minutes of preparation, when the expected stress level was lowest, and the measurement immediately after the completion of the mental arithmetic component of the test at the 13th minute, when the expected stress level was highest. The presumed cortisol decrease was measured from the completion of the mental arithmetic component of the test at the 13th minute to the 30th minute after test completion. We assessed the significance of the interaction between the measurement time during the stress test and the study group, using a mixed, between-within-subjects analysis of covariance. The analysis was adjusted for the preplanned covariates with possible confounding effects. We controlled possible confounding effects of age, sex, education, and work status by multivariable analysis. We analyzed the secondary outcome: differences in the baseline cortisol levels between FEP and controls using the analysis of covariance with adjustment for age, sex, education, and employment.

The normality of residuals was assessed by inspecting the histograms and Q-Q plots. We calculated η^2^ and Cohen’s d as the standardized effect sizes. To control the effect of multiple testing in secondary and tertiary outcomes, we used the Benjamini-Hochberg method with the false-discovery rate (FDR) set at ≤10% but considering all, the primary and tertiary outcome testing as well (study-wise correction). To assess the tertiary, explorative outcome, correlations of the cortisol levels and the results of the IPIP, SES, and PANSS in patients with FEP, we used the Spearman's correlation coefficient and a study-wise correction for multiple testing. The level of statistical significance was set at *P* < 0.05, always in two-tails tests, and all confidence intervals at 95%. The data analysis was performed with the R Core Team (2014), a language and environment for statistical computing (34).

## RESULTS

We enrolled 53 patients with FEP and 63 controls. FEP patients were more often men, somewhat older, less educated, and more often unemployed than controls (Table 1). Baseline cortisol measured before the stress test was higher in FEP than in controls (means [SD] for FEP and HC were 13.3 [6.49] nmol/L and 10.0 [4.21] nmol/L, respectively). This difference of 33% relative to the concentration in the control group was significant both in the bivariable analysis (F (1.114) = 11.3; *P* = 0.001; η2 = 0.09; d = 0.63; FDR<10%) and after adjustment for age, sex, education, and employment (F(1,109) = 9.4; *P* = 0.003; η^2^ = 0.08; d = 0.59; FDR<10%).

**Figure 1:**
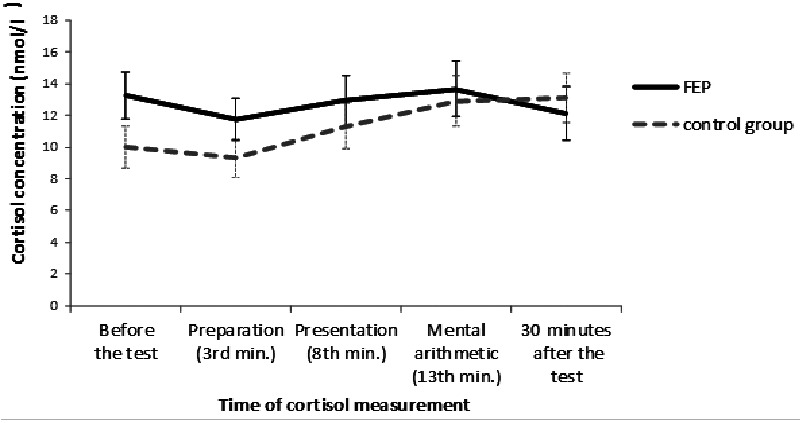
Change in salivary cortisol concentration (nmol/L) from test preparation (third min.) to 30 minutes after the test. Abbreviations: FEP – first-episode psychosis.

The change of cortisol level between the lowest value measured at the third minute after the participant preparation and the highest value measured after the stress test at the 13th minute was significantly smaller in FEP patients than in controls (Table 2, Figure 1). In the FEP group, cortisol increased for a mean of 19% (94%; CI 12%-27%), while in the control group it increased for a mean of 55% (95%; CI 37%-79%). The interaction of measurement and the study group was significant both in the bivariable analysis (F (1.114) = 6.0; *P* = 0.016; η^2^ = 0.05; d = 0.46) and after the adjustment for age, sex, education, and employment (F (1.110) = 4.7; *P* = 0.032; η^2^ = 0.04; d = 0.41). The lowering of cortisol during the 30 minutes after the stress test completion was not significantly different between the FEP and control group after the Benjamini-Hochberg correction with FDR≤10% (Table 3, Figure 1).

**Table 2:** Change in salivary cortisol concentration (nmol/L) from the preparation (third min.) to after the test (13th min.). Data are presented as mean (standard deviation) in unadjusted and mean (95% confidence interval) in adjusted analysis*

	After the preparation (third min.)		After the test (13th min.)		Δ	CI_95%_	Δ%	CI_95%_, %	η^2^ d	p
Unadjusted										
FEP	11.8	(5.53)	13.7	(6.66)	1.9	1.0-2.8	19	12-27	0.05	0.016
Control group	9.3	(4.16)	12.9	(5.97)	3.6	2.7-4.6	55	37-79	0.46	
Adjusted†										
FEP	11.8	(10.4-13.1)	13.7	(11.9-15.4)	1.9	0.8-3.0	19	0-39	0.04	0.032
Control group	9.3	(8.1-10.5)	12.9	(11.3-14.5)	3.6	2.6-4.6	55	38-73	0.41	

*Abbreviations: FEP – first episode psychosis; Δ – mean of absolute differences between the first and second measurement; Δ% – mean of relative differences calculated as the absolute difference divided by the baseline value; η^2^ – standardized effect size partial Eta squared; d – standardized effect size Cohen’s d; p –statistical significance of the interaction between time of measurement and the study group calculated using mixed between-within analysis of variance in unadjusted and analysis of covariance in adjusted analysis.

†Means were adjusted for age, sex, education, and employment status.

**Table 3:** Change in salivary cortisol concentration (nmol/L) from 13 to 30 min. after the test. Data are presented as mean (standard deviation) in unadjusted and mean (95% confidence interval) in adjusted analysis*

Unadjusted	After the test (13th min.)		30th min. after the test		Δ	CI_95%_	Δ%	CI_95%,_ %	η^2^ d	p	B-H
FEP	13.7	(6.66)	12.4	(5.90)	-1.2	-2.3 to-0.33	-6	-13-0	0.02	0.108	
Control group	12.9	(5.97)	12.9	(6.43)	-0.3	-1.0-1.1	3	-5-12	0.29		
Adjusted†											
FEP	13.7	(11.9-15.4)	12.2	(10.5-13.9)	-1.5	-2.6 to -0.4	-9	-18-0	0.04	0.034	n.s.
Control group	12.9	(11.3-14.5)	13.1	(11.6-14.7)	0.2	-0.8-1.2	5	0-13	0.41		

*Abbreviations: FEP – first episode psychosis; Δ – mean of absolute differences between the first and second measurement; Δ% – mean of relative differences calculated as the absolute difference divided by the baseline value; η^2^ – standardized effect size partial Eta squared; d – standardized effect size Cohen’s d; p –statistical significance of the interaction between time of measurement and the study group calculated using mixed between-within analysis of variance in unadjusted and analysis of covariance in adjusted analysis; B-H – statistically significant secondary outcomes at 10% false discovery rate calculated using Benjamini-Hochberg method; n.s. – not significant at FDR≤10%.

†Means were adjusted for age, sex, education, and employment status.

In patients, only baseline cortisol levels significantly positively correlated with negative and positive PANSS subscales, and emotional stability IPIP subscale, with one exception. However, in controls salivary cortisol levels positively correlated with emotional stability IPIP subscale, and negatively with SES and intellect/imagination IPIP subscale, in almost all measurements (Table 4).

**Table 4:** Correlations of salivary cortisol and tests’ results*

	Before the test		Preparation (third min.)		Presentation (eighth min.)		Mental arithmetic (13th min.)		30th min. after the test	
	ρ	p	ρ	p	ρ	p	ρ	p	ρ	p
FEP (n = 53)										
IPIP										
Extraversion	-0.06	0.697	-0.09	0.540	-0.05	0.709	-0.01	0.943	0.07	0.618
Agreeableness	-0.25	0.080	-0.22	0.129	-0.14	0.339	-0.03	0.863	0.11	0.444
Conscientiousness	0.28	0.046	0.15	0.310	0.03	0.839	0.10	0.481	0.18	0.217
Emotional stability	0.40	0.004†	0.40	0.003†	0.27	0.056	0.27	0.060	0.13	0.365
Intellect/Imagination	-0.16	0.277	-0.19	0.179	-0.20	0.162	-0.24	0.096	-0.21	0.134
SES	-0.24	0.089	-0.29	0.041	-0.20	0.160	-0.19	0.193	-0.01	0.966
Total PANSS score	0.37	0.006†	0.26	0.066	0.20	0.156	0.10	0.495	0.24	0.087
PANSS subscales										
positive symptoms	0.36	0.008†	0.24	0.091	0.19	0.169	0.08	0.590	0.20	0.158
negative symptoms	0.27	0.055	0.16	0.246	0.08	0.566	-0.01	0.926	0.14	0.308
general symptoms	0.34	0.012†	0.26	0.058	0.22	0.114	0.15	0.285	0.24	0.085
Control group (n = 63)										
IPIP										
Extraversion	0.12	0.365	0.11	0.390	0.12	0.371	0.03	0.836	0.00	0.985
Agreeableness	-0.22	0.089	-0.21	0.103	-0.23	0.072	-0.21	0.098	-0.14	0.262
Conscientiousness	-0.11	0.392	-0.20	0.121	-0.10	0.434	0.00	0.974	0.01	0.934
Emotional stability	0.31	0.014†	0.27	0.032	0.36	0.004†	0.32	0.011†	0.35	0.005†
Intellect/Imagination	-0.35	0.006†	-0.34	0.008†	-0.36	0.004†	-0.30	0.017†	-0.35	0.006†
SES	-0.33	0.008†	-0.35	0.005†	-0.32	0.010†	-0.21	0.092	-0.28	0.024†

*Abbreviations: ρ – Spearman's coefficient of rank correlation; p – statistical significance of the correlation coefficient, PANSS – The Positive and Negative Syndrome Scale for Schizophrenia; IPIP – The International Personality Item Pool; SES – Rosenberg Self-Esteem Scale.

†Statistically significant at FDR≤10% using a Benjamini-Hochberg method.

## DISCUSSION

In line with our hypothesis, we found that the patients with FEP had higher baseline levels of salivary cortisol and a blunted cortisol increase in response to psychosocial stressor compared with controls, but the groups did not differ in cortisol decrease after the peak activity. This is compatible with the results by Pruessner et al (16), who found significantly lower cortisol levels during the Trier Social Stress Test among persons at ultra-high risk for psychosis compared with controls. It is also compatible with a recent study among patients with FEP, which reported a blunted cortisol response to stressful activities, both on and off medication, compared with controls, although with no differences in baseline salivary cortisol levels (18). Interestingly, these findings are in line with studies among patients with posttraumatic stress disorder, who had increased cortisol levels and a lack of cortisol response during stress (20,21). A similar pattern was observed in healthy individuals with high trait anxiety (22), suggesting that high trait anxiety may be associated with an inability to respond to acute stress stimuli with adequate hormone release. The common feature in these disorders is unresolved early-life trauma, which produces chronic posttraumatic stress disorder, increases the vulnerability to stress, and eventually develops into other psychiatric disorders depending upon a variety of factors. Indeed, psychiatric disorders to a high degree share genetic and environmental roots, and the comorbidity of psychiatric disorders in clinical practice is very high, indicating common pathogenetic mechanisms (35). Thus, the question posed beyond psychotic illness is the effect of lifetime traumatic experiences on blunted neuroendocrine stress response, also observed in a recent publication (17). Since the effects of early trauma and the HPA axis adaptation over time are complex, different alterations of stress responses have been proposed as results of traumatic experiences, including elevated cortisol levels (36), dampened cortisol levels (37), and both increased (38) and blunted (39) stress-induced activation of sensitive brain regions.

However, a study by Seitz et al with a similar design and sample size to our study reported a different main result (17). They found that patients with FEP showed significantly lower cortisol levels throughout the afternoon testing period, but with no difference in the cortisol response to the Trier Social Stress Test compared with controls (17). While these differences may arise from the differences in the samples unrelated to FEP but associated with baseline cortisol, such as smoking or sex, they may also reflect the effect of FEP phase (acute, sub-acute) or medications. This phase-specific effect may be even more evident when it comes to the discrepancies between different studies in baseline salivary cortisol among patients with FEP, as we found higher baseline salivary cortisol levels in patients with more severe psychopathology, especially with positive and general psychotic symptoms. This is compatible with another study, in which the severity of acute psychotic symptoms correlated with the hyperactivity of HPA axis. Increased cortisol levels can lead to the sensitization of dopaminergic response to stress, which can cause an excessive dopamine release and psychotic symptoms (6), while in turn high dopamine levels produce psychotic experience, creating a vicious cycle.

The administration of antipsychotic treatments, which all act as dopamine receptor antagonists, may also influence cortisol levels. Zhang et al (40) found decreased baseline blood cortisol with the administration of risperidone and haloperidol in patients with schizophrenia, a finding that also correlated with a decrease in negative psychotic symptoms. Although blood baseline cortisol may not be an accurate measure, it is still worth investigating. Another question is whether antipsychotics administration may influence salivary cortisol response to stressors. Antipsychotics, but also other psychotropic drugs, such as antidepressants, alter HPA activity, but the extent and the direction of change is not well understood (11). To our knowledge, there are still no data regarding the influence of antipsychotics on the salivary cortisol response to stressors among patients with FEP. However, it was found that aripiprazole in the water blunts the stress response of exposed fish in a concentration ten times lower than the environmental concentration (41). Nonetheless, it is very difficult to separate the effects of medication and the effects of the illness *per se* in patients who are in the sub-acute phase.

Interestingly, Vaessen et al (18) found no differences between patients on and off medication, which possibly indicates that psychosis itself can contribute to a blunted stress response. Regardless whether the cause of decreased dopamine in the sub-acute phase is illness-associated or medication-induced (42), dopamine decrease may interfere with the activation of CRF, a major HPA-axis activator in stress response (39). This could possibly contribute to a blunted stress response in patients with psychosis or other psychiatric disorders. Interestingly, decreased general cortisol levels during the psychosocial stress test were associated with less active coping and lower self-esteem in patients with FEP but not in controls (17). Our results were partially concordant, as low cortisol levels were observed with higher self-esteem and high emotional stability, but only in controls. As this result was only exploratory, we will not discuss it further, apart from acknowledging the role of personality dimensions and self-esteem as possible mediators of cortisol response to psychosocial stressors unrelated to psychosis.

The strengths of this study are a relatively large sample of patients with FEP, homogenous in terms of age, diagnosis, and of phase of illness. However, the study also has several limitations. First, as we chose a consecutive sample of patients with FEP and a convenient control sample, the study may suffer from sample bias and limited representativeness of the targeted populations. Second, we cannot guarantee that the participants adhered to the protocol for saliva collection in terms of drink, food, and tobacco consumption although we relied on participants' self-reports. Other factors (eg, cannabis use) may affect the levels of salivary cortisol (43). Although we excluded individuals with addictions to psychoactive substances, these data were obtained by anamnesis only, without the confirmation using urine/ blood screening tests. Thus, we cannot exclude the unreported (occasional) use of cannabis among study participants, given the relatively high percentage of persons with FEP or controls who occasionally use marijuana.

While many alterations in the patients with FEP could be linked to alterations in stress response, a relatively small number of studies focused specifically on psychosocial stress in FEP. Our study supported the hypothesis that patients with FEP showed higher baseline levels of salivary cortisol, and an attenuated stress response to psychosocial stressor compared with healthy controls, suggesting that their vulnerability to stress arises from the decreased adaptive ability of cortisol to increase in response to stressors. Although these results are interesting and it seems reasonable that stress-coping strategies should be included in patients’ treatment plan, it is yet unclear whether these findings result from the specific effects of psychosis or other factors, including medication. Thus, we plan to follow-up these patients over a long period and analyze the influence of the mentioned factors.
